# Supplementary material for: Structural basis for Dicer-like function of an engineered RNase III variant and insights into the reaction trajectory of two-Mg2+-ion catalysis
Source: RNA Biol. 2022 Jul 13;19(1):908–15. doi: 10.1080/15476286.2022.2099650 (PMC9291653; doi:10.1080/15476286.2022.2099650)
Supplement: Supplemental Material [file KRNB_A_2099650_SM4127.zip › EcEEQ_v13_manuscript.pdf]

NIH Publishing Agreement & Manuscript *Cover Sheet*

By signing this Cover Sheet, the Author, on behalf of NIH, agrees to the provisions set out below, which **modify and supersede,** solely with respect to NIH, any conflicting or ambiguous provisions that are in the Publisher's standard copyright agreement (the "Publisher's Agreement"). NIH and Author accept only those provisions of the Publisher's Agreement that are consistent with the provisions herein, and only to the extent that they are directly related to the disposition of copyrights or duties of the parties that are directly related to the subject "Work." If a Publisher's Agreement is attached, execution of this Cover Sheet constitutes an execution of the Publisher's Agreement, subject to the provisions and conditions of this Cover Sheet.

1. **Indemnification.** No Indemnification or "hold harmless" obligation is provided by either party.

2. **Governing Law.** This agreement will be governed by the law of the court in which a claim is brought.

3. **Copyright.** Author's contribution to the Work was done as part of the Author's official duties as a NIH employee and is a Work of the United States Government. Therefore, copyright may not be established in the United States. 17 U.S.C. § 105. If Publisher intends to disseminate the Work outside of the U.S., Publisher may secure copyright to the extent authorized under the domestic laws of the relevant country, subject to a paid-up, nonexclusive, irrevocable worldwide license to the United States in such copyrighted work to reproduce, prepare derivative works, distribute copies to the public and perform publicly and display publicly the work, and to permit others to do so.

4. **No Compensation.** No royalty income or other compensation may be accepted for work done as part of official duties. The author may accept for the agency a limited number of reprints or copies of the publication.

5. **NIH Representations.** NIH represents to the Publisher that the Author is the sole author of the Author's contribution to the Work and that NIH is the owner of the rights that are the subject of this agreement; that the Work is an original work and has not previously been published in any form anywhere in the world; that to the best of NIH's knowledge the Work is not a violation of any existing copyright, moral right, database right, or of any right of privacy or other intellectual property, personal, proprietary or statutory right; that where the Author is responsible for obtaining permissions or assisting the Publishers in obtaining permissions for the use of third party material, all relevant permissions and information have been secured; and that the Work contains nothing misleading, obscene, libelous or defamatory or otherwise unlawful.

6. **Disclaimer.** NIH and the Author expressly disclaim any obligation in Publisher's Agreement that is not consistent with the Author's official duties or the NIH mission. NIH and the Author do not disclaim obligations to comply with a Publisher's conflict of interest policy so long as, and to the extent that, such policy is consistent with NIH's own conflict of interest policies.

7. **For Peer-Reviewed Papers to be Submitted to PubMed Central.** The Author is a US government employee who must comply with the NIH Public Access Policy, and the Author or NIH will deposit, or have deposited, in NIH's PubMed Central archive, an electronic version of the final, peer-reviewed manuscript upon acceptance for publication, to be made publicly available no later than 12 months after the official date of publication. PubMed Central may tag or modify the work consistent with its customary practices.

The NIH Deputy Director for Intramural Research, Michael Gottesman, M.D., approves this publishing agreement and maintains a single, signed copy of this text for all works published by NIH employees, contractors and trainees who are working at the NIH. No additional signature from Dr. Gottesman is needed.

Author's name: Sudhaker Dharavath, Gary X. Shaw and Xinhua Ji

Author's Institute or Center: National Cancer Institute

Name of manuscript/work: Structural basis for Dicer-like function of an engineered RNase III variant and insights into the reaction trajectory of two-Mg2+-ion catalysis

Name of publication: RNA Biology

Is Publisher's Agreement attached? ☐ yes ☑ no

Xinhua Ji

Author's signature

May 9, 2022

Date

NIH OGC July 2008

Structural basis for Dicer-like function of an engineered RNase III variant and insights into the reaction trajectory of two-Mg^{2+}-ion catalysis

Sudhaker Dharavath, Gary X. Shaw, and Xinhua Ji*

Center for Structural Biology, National Cancer Institute, Frederick, MD 21702, USA

* To whom correspondence should be addressed. Tel: 1-301-846-5035; Fax: 1-301-846-6073;

Email: jix@mail.nih.gov

E-MAIL

Sudhaker Dharavath: sudhaker.dharavath@nih.gov

Gary X. Shaw: shawg@mail.nih.gov

Xinhua Ji: jix@mail.nih.gov

ORCID

Sudhaker Dharavath: 0000-0002-5973-0214

Gary X. Shaw: 0000-0002-2510-3538

Xinhua Ji: 0000-0001-6942-1514

ABSTRACT

The RNase III family of dsRNA-specific endonucleases is exemplified by prokaryotic RNase III and eukaryotic Rnt1p, Drosha, and Dicer. Structures of *Aquifex aeolicus* RNase III (AaRNase III) and *Saccharomyces cerevisiae* Rnt1p (ScRnt1p) show that both enzymes recognize substrates in a sequence-specific manner and propel RNA hydrolysis by two-Mg^{2+}-ion catalysis. Previously, we created an *Escherichia coli* RNase III variant (EcEEQ) by eliminating the sequence specificity via protein engineering and called it bacterial Dicer for the fact that it produces heterogeneous small interfering RNA cocktails. Here, we present a 1.8-Å crystal structure of a postcleavage complex of EcEEQ, representing a reaction state immediately after the cleavage of scissile bond. The structure not only establishes the structure-and-function relationship of EcEEQ, but also reveals the functional role of a third Mg^{2+} ion that is involved in RNA hydrolysis by bacterial RNase III. In contrast, the cleavage site assembly of ScRnt1p does not contain a third Mg^{2+} ion. Instead, it involves two more amino acid side chains conserved among eukaryotic RNase IIIs. We conclude that the EcEEQ structure (this work) represents the cleavage assembly of prokaryotic RNase IIIs and the ScRnt1p structure (PDB: 4OOG), also determined at the postcleavage state, represents the cleavage assembly of eukaryotic RNase IIIs. Together, these two structures provide insights into the reaction trajectory of two-Mg^{2+}-ion catalysis by prokaryotic and eukaryotic RNase III enzymes.

KEYWORDS

RNase III; Rnt1p; Drosha; Dicer; dsRNA; RNA hydrolysis; two-Mg^{2+}-ion catalysis; postcleavage complex

Introduction

Discovered in 1968, *Escherichia coli* RNase III (EcRNase III) is the founding member of the RNase III family of dsRNA-specific endoribonucleases found in all kingdoms of life (1,2). Representative members of the family include bacterial RNase III, yeast Rnt1p, human Drosha, and human Dicer. A common type of substrate for all RNase III enzymes is stem-loop RNA. Also known as hairpin RNA, stem-loop RNA is an essential secondary structure of primary importance (3). A less common type of substrate for some RNase IIIs is long duplex RNA. Among the four representative family members, Rnt1p and Drosha process stem-loop RNAs only, whereas RNase III and Dicer also process long dsRNAs. In a successive manner, the processing starts from one end of a long dsRNA and produces small duplex RNAs (4-6). Dicer typically measures 22 nucleotides for cleavage, producing small interfering RNAs (siRNAs) (Figure 1A). In contrast, RNase III typically measures 11 nucleotides for cleavage, resulting in small duplex RNAs of half the length of siRNAs (Figure 1B) (7). In addition to this end-in manner of processing, long dsRNA can also be processed by RNase III in an inside-out scheme, where multiple RNase III molecules bind consecutively to a long dsRNA and cleave the substrate simultaneously (8). Since the distance between consecutive active centers of adjacent RNase III molecules is 22 nucleotides, the inside-out processing produces siRNA-like small duplex RNAs, indistinguishable from the siRNAs produced by Dicer (Figure 1B). This mechanism has been observed for EcRNase III under special conditions (9).

Since the discovery of EcRNase III, tremendous progress has been made in studies of bacterial enzymes (10,11). Genetic and functional studies have been performed mainly with EcRNase III, whereas structural and mechanistic studies have been carried out mainly with *Aquifex aeolicus* RNase III (AaRNase III). Crystal structures of AaRNase III have been

determined at several distinct catalytic states, providing insights into the mechanisms of substrate recognition, scissile bond selection, two-Mg^{2+}-ion catalysis, phosphoryl transfer, and product release (6,11-13). In addition, a hypothesis that the E38A mutant of EcRNase III (EcE38A) could promote the inside-out processing of long dsRNA led to the discovery of a reagent for the preparation of siRNA cocktails to be used in gene silencing studies (Figure 1C) (14). It offers an economic way for siRNA cocktail preparation since making EcE38A costs much less than the production of human Dicer. Residue E38 is conserved among bacterial RNase IIIs and is located on the RNA-contacting surface distant from the cleavage site. Bacterial RNase III functions as a dimer, and thus removing two negatively charged side chains between the protein and RNA promotes consecutive binding and cleavage of long dsRNA by multiple RNase III dimers (Figure 1C).

By next generation sequencing, we previously demonstrated that both EcRNase III and AaRNase III cleave long dsRNAs at preferred sites (7). The AaRNase III structures suggest that the specificity for a guanine nucleotide at the +3 position near the scissile bond could be eliminated by an alanine substitution at Q165 of EcRNase III. Like E38, residue E65 of EcRNase III is also located on the RNA-contacting surface. Therefore, the E65A mutation in EcRNase III could also assist in promoting the inside-out mechanism. These two predictions prompted us to create a triple mutant (E38A/E65A/Q165A) of EcRNase III (EcEEQ, Figure 1C), which indeed produces heterogeneous siRNA cocktails (7). Therefore, we call this EcRNase III variant bacterial Dicer. It is remarkable that following distinct mechanisms, human Dicer (end-in) and bacterial Dicer (inside-out) produce indistinguishable products (Figure 1). Here, we report the crystal structure of EcEEQ in complex with a stem-loop RNA (RNA6) at 1.80-Å resolution

(EcEEQ:RNA6), showing how the E38A and E65A mutations stabilize the protein:RNA complex and how the Q165A mutation eliminates the sequence specificity of EcRNase III.

To enhance substrate specificity and catalytic efficiency, two-Mg^{2+}-ion catalysis is used by all RNA and DNA polymerases, and most nucleases and recombinases (15). The two-Mg^{2+}-ion catalysis by RNase III was established by structures of AaRNase III and ScRnt1p, in the form of a postcleavage complex determined at a stage immediately after the cleavage of the scissile bond (12,16). Intriguingly, the EcEEQ:RNA6 structure reveals the functional role of a third Mg^{2+} ion that is involved in the mechanism of two-Mg^{2+}-ion catalysis by bacterial RNase III.

Results and discussion

The EcEEQ:RNA6 is highly homologous to the postcleavage complex of AaRNase III

We started working on EcRNase III in 1996, but we were not able to crystallize the protein, either full-length or truncated. In parallel with EcEEQ, we performed crystallization trials again with wild-type EcRNase III in this study, but only the EcEEQ:RNA6 complex crystalized, underscoring the significant impact of removing four negatively charged amino acid side chains from RNA-contacting surface of the protein on the stability of the protein:RNA complex. Solving the phase problem by molecular replacement (MR), we first utilized the AaRNase III:RNA6 structure, a protein-product complex containing one Mg^{2+} ion in each cleavage site (6), as the search model, which resulted in multiple solutions of low log-likelihood gain (LLG) and final translation function Z (TFZ) scores (top solution: LLG = 71; TFZ = 6.0). Then, we tested the postcleavage complex of AaRNase III (AaRNase III:RNA9), containing two Mg^{2+} ions in each cleavage site (12), which resulted in a unique solution of much higher LLG and TFZ scores (LLG = 445; TFZ = 15.9). Hence, the EcEEQ:RNA6 structure resembles the postcleavage

complex of AaRNase III, indicating that the EcEEQ:RNA6 complex is also a postcleavage complex. The structure is indeed a postcleavage complex with an additional feature. It reveals that a third Mg^{2+} ion is integrated in the cleavage site assembly of two-Mg^{2+}-ion catalysis by bacterial RNase IIIs.

The EcEEQ:RNA6 structure is schematically illustrated in Figure 2A. The complex is composed of two EcEEQ subunits (each containing 226 amino acid residues), two RNA6 molecules (each containing 28 nucleotide residues in the form of a hairpin with a 4-nucleotide capping loop and a 2-nucleotide 3′ overhang), six Mg^{2+} ions, 418 water oxygen atoms, and several other ions and molecules from the solvent and cryoprotectant. All three Mg^{2+} ions in the cleavage site of each subunit are well defined with full occupancy (Figure 2B). Each EcEEQ molecule contains a specialized endonuclease domain (RIIID, residues 1-147) and a dsRNA-binding domain (dsRBD, residues 156-226, Figure 2C). The root-mean-square deviation (RMSD) between the two EcEEQ subunits is 0.17 Å for 196 out of 226 pairs of Cα atoms, and the RMSD between the two RNA6 molecules is 0.14 Å for 587 out of 598 pairs of atoms, underscoring the highly symmetric nature of the EcEEQ:RNA6 complex (Figure 2A).

The EcEEQ:RNA6 and AaRNase III:RNA9 structures are highly homologous. The EcEEQ structure exhibits 15 secondary structure elements, of which 13 are shared with AaRNase III (Figure 2C). Eleven out of the 13 shared secondary structure elements are of the same length in the two structures, whereas two (α7 and α8) are one residue longer in AaRNase III. Superposition of the EcEEQ:RNA6 and AaRNase III:RNA9 complexes shows that the two structures align very well (Figure 3A). The RMSD for 404 out of 417 pairs of Cα atoms between the two RNase III dimers is 1.7 Å. Including RNA, the RMSD for 382 out of 458 pairs of Cα

and P atoms is 1.6 Å. The high homology of the two structures offers the convenience of using just the numbering scheme of the focus, i.e., the residue numbers in EcRNase III.

Structural basis for Dicer-like function of EcEEQ

The first glimpse at the RNase III active site was provided by our structure of *A. aeolicus* RIIID dimer 33 years after the discovery of the enzyme (17,18). The structure showed that the dimerization of RIIID creates a catalytic valley about 20 Å wide and 50 Å long, which can accommodate a dsRNA substrate. Five years later, our AaRNase III:RNA6 structure showed that the proposed catalytic valley is indeed the binding site of a dsRNA substrate (6). In the middle of the valley are located two cleavage sites, each featuring a cluster of four negatively charged side chains (E41, D45, D114, and E117) (12). At each end of the valley, two more negatively charged side chains (E38 and E65) are located. These negatively charged side chains are conserved among bacterial RNase III enzymes.

As depicted in Figure 3, panels A and B, residues E38 and E65 are located on the RNA-contacting surface. Therefore, the alanine mutation of both E38 and E65 stabilizes the resulting protein:RNA complex and thereby promotes the inside-out processing of long dsRNAs. This structural implication was validated by *in vitro* cleavage products analysis (7). The third mutation in EcEEQ is Q165A that is critical for the production of heterogeneous siRNA cocktails. As shown in Figure 3C, the Q165 side chain forms three hydrogen bonds with the +3G nucleotide, two with the base and one with the O2′-hydroxyl group (12). The length of these hydrogen bonds ranges between 2.9 and 3.1 Å. Hence, the Q165A mutation eliminates these strong hydrogen bonds and thereby abolishes its specificity for the +3G nt. This structural implication was also validated by *in vitro* cleavage products analysis (7). The loss of three

hydrogen bonds between the protein and RNA results in a less stable protein:RNA complex. This negative impact on the stability is offset by the overwhelming stabilization effect of the E38A and E65A mutations as demonstrated previously by product analysis of *in vitro* cleavage (7).

As mentioned above, the RNA-contacting surface of the catalytic valley is coated by a total of 12 negatively charged side chains. The E38A and E65A mutations neutralize four of them. The remaining eight are catalytic side chains, forming two cleavage sites in the catalytic valley. Interactions between these catalytic side chains and dsRNA are bridged by catalytic Mg^{2+} ions that mitigate electrostatic repulsion between the enzyme and the RNA. No other negatively charged side chains exist between the catalytic valley and the bond dsRNA. Therefore, the impact of E38A and E65A mutations on the stability of the protein:RNA complex is profound. Taken together, these features revealed by the EcEEQ:RNA6 and AaRNase III:RNA9 complexes are structural basis for the function of EcEEQ, the bacterial Dicer that is most suitable for producing heterogeneous siRNA cocktails.

The EcEEQ:RNA6 represents the postcleavage state of RNA hydrolysis by bacterial RNase III

The RIIID dimer hydrolyzes both strands of dsRNA simultaneously with two identical cleavage sites. In each cleavage site of the EcEEQ:RNA6 structure, three Mg^{2+} ions are well defined and fully occupied. As depicted in Figure 4A, these Mg^{2+} ions (MgA, MgB and MgC) are "organizers" of the cleavage site assembly by coordinating with four catalytic side chains (E41, D45, D114, and E117), three nucleotide residues (R-1, R 0, and R+1), and eight water molecules around the scissile bond. MgA coordinates with two oxygen atoms of the scissile phosphate group (R 0), two oxygen atoms of the E41 and E117 side chains, and two oxygen atoms of water molecules. MgB coordinates with one oxygen atom of the scissile phosphate group (R 0), the

oxygen atom of 3′-OH group (R-1), two oxygen atoms of the D45 and E117 side chains, and two oxygens of water molecules. MgC coordinates with one oxygen of the scissile phosphate group (R 0), one phosphate oxygen of the R+1 nucleotide, and four oxygen atoms of water molecules. Among the four catalytic side chains, only D114 does not coordinate with Mg^{2+} directly. Bridging negatively charged components, the three Mg^{2+} ions make the cleavage site assembly as compact as needed for catalysis.

The compactness of the assembly can be measured with two contact distances (CDs), one between MgA and MgB ($CD^{MgA,MgB}$) and the other between 3′-oxygen of nucleotide R-1 and phosphorus of nucleotide R 0 ($CD^{O3′,P}$). The phosphorus of nucleotide R 0 represents the scissile phosphate group after RNA hydrolysis. Before the reaction, it is covalently bonded to the 3′-oxygen of nucleotide R-1. In the EcEEQ:RNA6 structure, the $CD^{O3′,P}$ measures 2.9 Å (Figure 4A), which is smaller than the sum of van der Waals (vdW) radii of O (1.52 Å) and P (1.80 Å) (19). As shown in Figure 4B, the cleavage site assembly in the AaRNase III:RNA9 structure exhibits two distinct conformations, a conformation of 25% occupancy (minor-AaRNase III:RNA9) and a conformation of 75% occupancy (major-AaRNase III:RNA9) (12). The $CD^{O3′,P}$ in minor-AaRNase III:RNA9 measures 3.0 Å, indicating that 25% of the population represents the postcleavage state, whereas it measures 3.5 Å in major-AaRNase III:RNA9, indicating that 75% of the population represents a state of product release. Whereas two Mg^{2+} ions were observed in minor-AaRNase III:RNA9, three Mg^{2+} ions were observed in major-AaRNase III:RNA9. Neither MgC nor its four coordination water molecules are observed in minor-AaRNase III:RNA9 (Figure 4B). It has been previously shown that $CD^{MgA,MgB}$ is about 3.5 Å at the intermediate state for the two-Mg^{2+}-ion catalysis by either RNase H1 or DNAPη (20,21). The $CD^{MgA,MgB}$ in the EcEEQ:RNA6 structure is 3.7 Å (Figure 4A), suggesting that the

EcEEQ:RNA6 structure represents the postcleavage state immediately after the intermediate state of RNA hydrolysis.

Structural insights into two-Mg^{2+}-ion catalysis by bacterial and yeast RNase III enzymes

Nucleophilic attack on phosphorus could produce a relatively long-lived pentacovalent intermediate. And all phosphoryl transfer reactions in DNA and RNA involve such an intermediate and inversion of the stereo configuration at the phosphorus (15). Based on the EcEEQ:RNA6 structure, models of reaction intermediate and precleavage complex could be readily derived by adjusting the torsion angles along the C4′-C5′-O5′-P-O1P chain and breaking or making the P-O bonds. At the postcleavage state, a scissile-phosphate oxygen is coordinated with both MgA and MgC (Figure 5A). This special oxygen atom is in fact the nucleophilic water oxygen at the intermediate (Figure 5B) and precleavage states (Figure 5C). It appears that MgC plays three important roles in catalysis. First, MgC teams with MgA and MgB to optimize the cleavage site geometry for the formation of the pentacovalent intermediate (Figure 5B). Second, MgC synergizes with MgA to activate the nucleophilic water molecule (Figure 5C). Third, MgC facilitates the nucleophilic attack and subsequent electron transfer (Figure 5B). Coordinated with the scissile phosphate, MgC has been observed not only at the postcleavage state (EcEEQ:RNA6, Figure 4A), but also at two distinct snapshots during product release, one after the scissile phosphate moves away from the cleavage site (major-AaRNase III:RNA9, Figure 4B) and the other after the scissile hydroxyl also moves away from the cleavage site (12). We predict that all prokaryotic RNase IIIs employ the third Mg^{2+} ion to assist two-Mg^{2+}-ion catalysis of RNA hydrolysis.

Unlike bacterial RNase IIIs that use four catalytic side chains (E1, D2, D3, and E4) in each event of two-Mg^{2+}-ion catalysis, yeast Rnt1p uses two more (N5 and K6) that are conserved among eukaryotic RNase IIIs (Figure 5D). As revealed by the Rnt1p postcleavage structure, the side chains of E1, D2, D3, and E4 in Rnt1p assume identical positioning as in bacterial RNase IIIs, whereas those of N5 and K6 are unique for eukaryotes (16). The side chain of N5 interacts with two water molecules and one oxygen of the 5′ phosphate while the ε-amino group of K6 side chain interacts with one oxygen of the 5′ phosphate and another from the carboxylic group of D3 (Figure 5E). As shown, in the presence of N5 and K6, the $CD^{MgA,MgB}$ is 3.6 Å at the postcleage state of Rnt1p. Like the MgC in the two-Mg^{2+}-ion catalysis of prokaryotic RNase IIIs, the N5 and K6 side chains also play three important roles. First, the K6 side chain recognizes the scissile phosphate group and teams with MgA and MgB to optimize the cleavage site geometry for the formation of the pentacovalent intermediate (Figure 5F). Second, K6 side chain synergizes with MgA to activate the nucleophilic water molecule (Figure 5G). Third, K6 and N5 side chains facilitate the nucleophilic attack and subsequent electron transfer (Figure 5F). Since K6 is not conserved in one RIIID among eukaryotic RNase III enzymes (8), we predict that all eukaryotic RNase IIIs employ the N5 and most also employ K6 side chains to assist two-Mg^{2+}-ion catalysis of RNA hydrolysis. In the presence of K6, the positively charged ε-amino group prevents a positively charged metal ion from binding at the MgC site. In the absence of K6, however, whether a third metal ion would bind at the MgC site remains to be elucidated.

Materials and methods

Protein expression and purification

The expression vector of EcEEQ was constructed and His_6-MBP tagged EcEEQ (His_6-MBP-EcEEQ) was overproduced in *E. coli* BL21(DE3) Codon Plus-RIL cells (ThermoFisher Scientific, Waltham, MA) as described (7) with limited modifications. Briefly, the cells were cultivated in Luria-Bertani (LB) broth containing 100 μg ml^{-1} ampicillin and 30 μg ml^{-1} chloramphenicol at 37°C, induced by the addition of isopropyl β-d-1-thiogalactopyranoside (IPTG) to a final concentration of 1 mM, and shaken for 4-6 hr at 37°C. The cells were harvested by centrifugation at 4000 g for 10 min at 4°C and lysed in 30 mM Tris (pH 7.4), 1 M NaCl, and 100 μl l^{-1} 2-mercaptoethanol by sonication at 45 kHz. After removal of insoluble cell debris by centrifugation at 12000 rpm for 30 min, the supernatant was filtered through a 0.45-μm cellulose acetate membrane and applied to a HisTrap FF column (GE Healthcare Life Sciences, Pittsburgh, PA). Equilibration and washing were performed using a buffer containing 30 mM Tris (pH 7.4), 1 M NaCl, 20 mM imidazole, and 100 μl l^{-1} 2-mercaptoethanol, and elution was carried out in 30 mM Tris (pH 7.4), 1 M NaCl, 400 mM Imidazole, and 100 μl l^{-1} 2-mercaptoethanol. Fractions containing His_6-MBP-EcEEQ were pooled. The His_6-MBP tag was removed by cleaving the fused protein with 0.5 mg ml^{-1} TEV protease (22) at a designed site in the linker and passing the digested protein through a reverse HisTrap FF column to yield the recombinant EcEEQ protein. The EcEEQ protein was further purified with a HiLoad (26/60) Superdex 200 size exclusion column (GE Healthcare Life Sciences). The final product, in 25 mM Tris (pH 7.4), 200 mM NaCl, and 100 μl l^{-1} 2-mercaptoethanol, was concentrated to 12 mg ml^{-1} (determined spectrometrically using a molar extinction coefficient of 14440 M^{-1} cm^{-1}), aliquoted, flash frozen in liquid nitrogen, and stored at -80°C.

Crystallization and X-Ray diffraction data collection

Previously, a total of 12 RNA oligos (RNA1 through RNA12) were used and/or observed in the structures of AaRNase III:RNA complexes (11). RNA6, a 28-nucleotide stem-loop RNA derived from a canonical substrate of EcRNase III (6), was purchased from Dharmacon RNA Technologies (Chicago, IL) for this study. Prior to crystallization, the protein and RNA were incubated at room temperature for 30 min in a solution consisting of 7.6 mg ml^{-1} EcEEQ, 0.4 mM RNA6, 50 mM $MgCl_2$, 300 mM NaCl, and 25 mM Tris-HCl (pH 7.4). The crystallization screening was carried out with a Mosquito crystallization robot (SPT Labtech Ltd., Hertfordshire, UK) by sitting drop vapor diffusion and the plates were incubated at 19±1°C. Micro crystals appeared after 3 days in drops containing the protein-RNA solution and an equal volume of well solution (25% PEG 3350 and 0.2 M KBr in 100 mM Hepes buffer, pH 7.5) and reached suitable size for X-ray diffraction after 1-2 weeks. The crystals were flash-frozen after being soaked briefly in a cryo-protection solution containing 75% (v/v) reservoir solution and 25% (v/v) ethylene glycol. X-ray diffraction data were collected at 100 K at the Southeast Regional Collaborative Access Team (SER-CAT) insertion device beamline 22 (22-ID) of the Advanced Photon Source, Argonne National Laboratory. The data was indexed, integrated, and scaled with the HKL3000 program suite (23). Data collection and processing statistics are summarized in Table 1.

Structure solution and Refinement

The structure of the EcEEQ:RNA6 complex was solved by molecular replacement (MR) using PHASER (24). As mentioned above, the search model was the AaRNase III:RNA9 structure (12) after solvent molecules and ions were removed. The sequence identity between AaRNase III and

EcEEQ is 33%. Each RNase III:RNA complex contains two RNase III and two RNA molecules. RNA9 contains 22 nucleotide residues. Therefore, 44 nucleotides are present in the AaRNase III:RNA9 search model, whereas 56 nucleotides are present in the EcEEQ:RNA6 structure. Nonetheless, the MR solution was unique with high LLG and TFZ scores (LLG = 445; TFZ = 15.9). Starting with the MR solution, the phases were improved with phenix_mr_rosetta (25) by density- and energy-guided model optimization and iterative model rebuilding. Although the current version of phenix_mr_rosetter did not work on nucleic acids structures, the phasing power of the EcEEQ protein were so strong that the difference Fourier electron density revealed the structure of two RNA6 molecules in their entirety. The initial RNA6 molecules that we built with COOT (26) were remodelled with ERRASER (Enumerative Real-Space Refinement ASsisted by Electron density under Rosetta) maintained at the ROSIE server (27,28). Further adjustment and refinement of the structure were carried out with COOT (26) and PHENIX (29). The quality of the final structure was validated on the Worldwide PDB (wwPDB) Validation Server (30). The structure refinement statistics are summarized in Table 1. Illustrations were prepared using PyMOL (Schrödinger, LLC.).

Based on the EcEEQ:RNA6 structure, model complexes for the intermediate and precleavage states of EcRNase III were derived by adjusting the torsion angles along the C4′-C5′-O5′-P-O1P chain and breaking or making the P-O bonds (12). Similarly, model complexes for the intermediate and precleavage states of ScRnt1p were derived based on the structure of its postcleavage complex (16). No adjustments were made to other components of the cleavage assemblies because the $CD^{MgA,MgB}$ values (3.6 or 3.7 Å) mimic that at the intermediate state (3.5 Å) observed for RNase H1 and DNAPη (20,21).

Acknowledgments

We thank Lan Jin and He Song for discussion and Joshua Rose and Alexander Wlodawer for reading the manuscript. X-ray diffraction data were collected at the SER-CAT 22-ID beamline at the Advanced Photon Source, Argonne National Laboratory.

Disclosure statement

The authors declare no conflict of interest.

Funding

This research was supported by the Intramural Research Program of the NIH, National Cancer Institute, Center for Cancer Research.

Data availability statement

The atomic coordinates and structure factors for the EcEEQ:RNA6 complex has been deposited with the Protein Data bank (PDB: 7R97).

References

1. Robertson, H.D., Webster, R.E. and Zinder, N.D. (1968) Purification and properties of ribonuclease III from *Escherichia coli*. *J. Biol. Chem.*, **243**, 82-91.
2. Nicholson, A.W. (2003) In Hannon, G. J. (ed.), *RNAi: A Guide to Gene Silencing*. Cold Spring Harbor Laboratory Press, Cold Spring Harbor, New York, pp. 149-174.
3. Svoboda, P. and Di Cara, A. (2006) Hairpin RNA: a secondary structure of primary importance. *Cell Mol. Life Sci.*, **63**, 901-908.
4. Court, D.L. (1993) In Belasco, J. G. and Brawerman, G. (eds.), *Control of Messenger RNA Stability*. Academic Press, New York, pp. 71-116.
5. Liu, Z., Wang, J., Cheng, H., Ke, X., Sun, L., Zhang, Q.C. and Wang, H.W. (2018) Cryo-EM Structure of Human Dicer and Its Complexes with a Pre-miRNA Substrate. *Cell*, **173**, 1191-1203 e1112.
6. Gan, J., Tropea, J.E., Austin, B.P., Court, D.L., Waugh, D.S. and Ji, X. (2006) Structural insight into the mechanism of double-stranded RNA processing by ribonuclease III. *Cell*, **124**, 355-366.
7. Jin, L., Song, H., Tropea, J.E., Needle, D., Waugh, D.S., Gu, S. and Ji, X. (2019) The molecular mechanism of dsRNA processing by a bacterial Dicer. *Nucleic Acids Res.*, **47**, 4707-4720.
8. Weinberg, D.E., Nakanishi, K., Patel, D.J. and Bartel, D.P. (2011) The inside-out mechanism of Dicers from budding yeasts. *Cell*, **146**, 262-276.
9. Yang, D., Buchholz, F., Huang, Z., Goga, A., Chen, C.Y., Brodsky, F.M. and Bishop, J.M. (2002) Short RNA duplexes produced by hydrolysis with *Escherichia coli* RNase III

mediate effective RNA interference in mammalian cells. *Proc. Natl. Acad. Sci. U.S.A.*, **99**, 9942-9947.

10. Nicholson, A.W. (2014) Ribonuclease III mechanisms of double-stranded RNA cleavage. *Wiley interdisciplinary reviews. RNA*, **5**, 31-48.

11. Court, D.L., Gan, J., Liang, Y.-H., Shaw, G.X., Tropea, J.E., Costantino, N., Waugh, D.S. and Ji, X. (2013) RNase III: Genetics and Function; Structure and Mechanism. *Annu. Rev. Genet.*, **47**, 405-431.

12. Gan, J., Shaw, G., Tropea, J.E., Waugh, D.S., Court, D.L. and Ji, X. (2008) A stepwise model for double-stranded RNA processing by ribonuclease III. *Mol. Microbiol.*, **67**, 143-154.

13. Gan, J., Tropea, J.E., Austin, B.P., Court, D.L., Waugh, D.S. and Ji, X. (2005) Intermediate states of ribonuclease III in complex with double-stranded RNA. *Structure (Camb)*, **13**, 1435-1442.

14. Xiao, J., Feehery, C.E., Tzertzinis, G. and Maina, C.V. (2009) E. coli RNase III(E38A) generates discrete-sized products from long dsRNA. *RNA*, **15**, 984-991.

15. Yang, W., Lee, J.Y. and Nowotny, M. (2006) Making and breaking nucleic acids: two-Mg2+-ion catalysis and substrate specificity. *Mol. Cell*, **22**, 5-13.

16. Liang, Y.H., Lavoie, M., Comeau, M.A., Abou Elela, S. and Ji, X. (2014) Structure of a eukaryotic RNase III postcleavage complex reveals a double-ruler mechanism for substrate selection. *Mol. Cell*, **54**, 431-444.

17. Blaszczyk, J., Tropea, J.E., Bubunenko, M., Routzahn, K.M., Waugh, D.S., Court, D.L. and Ji, X. (2001) Crystallographic and modeling studies of RNase III suggest a mechanism for double-stranded RNA cleavage. *Structure*, **9**, 1225-1236.

18. Zamore, P.D. (2001) Thirty-three years later, a glimpse at the ribonuclease III active site. *Mol. Cell*, **8**, 1158-1160.

19. Mantina, M., Chamberlin, A.C., Valero, R., Cramer, C.J. and Truhlar, D.G. (2009) Consistent van der Waals radii for the whole main group. *J Phys Chem A*, **113**, 5806-5812.

20. Nowotny, M. and Yang, W. (2006) Stepwise analyses of metal ions in RNase H catalysis from substrate destabilization to product release. *EMBO J.*, **25**, 1924-1933.

21. Batra, V.K., Beard, W.A., Shock, D.D., Krahn, J.M., Pedersen, L.C. and Wilson, S.H. (2006) Magnesium-induced assembly of a complete DNA polymerase catalytic complex. *Structure*, **14**, 757-766.

22. Kapust, R.B., Tözsér, J., Fox, J.D., Anderson, D.E., Cherry, S., Copeland, T.D. and Waugh, D.S. (2001) Tobacco etch virus protease: mechanism of autolysis and rational design of stable mutants with wild-type catalytic proficiency. *Protein. Eng.*, **14**, 993-1000.

23. Otwinowski, Z. and Minor, W. (1997) Processing of X-ray diffraction data collected in oscillation mode. *Methods Enzymol.*, **276**, 307-326.

24. McCoy, A.J., Grosse-Kunstleve, R. W., Adams, P. D., Winn, M. D., Storoni, L.C. & Read R.J. . (2007) Phaser crystallographic software. *J. Appl. Crystallogr.*, **40**, 658-674.

25. Terwilliger, T.C., Dimaio, F., Read, R.J., Baker, D., Bunkoczi, G., Adams, P.D., Grosse-Kunstleve, R.W., Afonine, P.V. and Echols, N. (2012) phenix.mr_rosetta: molecular replacement and model rebuilding with Phenix and Rosetta. *J Struct Funct Genomics*, **13**, 81-90.

26. Emsley, P. and Cowtan, K. (2004) Coot: model-building tools for molecular graphics. *Acta Crystallogr. D*, **60**, 2126-2132.

27. Chou, F.C., Sripakdeevong, P., Dibrov, S.M., Hermann, T. and Das, R. (2013) Correcting pervasive errors in RNA crystallography through enumerative structure prediction. *Nat Methods*, **10**, 74-76.

28. Sripakdeevong, P., Kladwang, W. and Das, R. (2011) An enumerative stepwise ansatz enables atomic-accuracy RNA loop modeling. *Proc. Natl. Acad. Sci. U.S.A.*, **108**, 20573-20578.

29. Adams, P.D., Afonine, P.V., Bunkoczi, G., Chen, V.B., Davis, I.W., Echols, N., Headd, J.J., Hung, L.W., Kapral, G.J., Grosse-Kunstleve, R.W. *et al.* (2010) PHENIX: a comprehensive Python-based system for macromolecular structure solution. *Acta Crystallogr. D*, **66**, 213-221.

30. Gore, S., Sanz Garcia, E., Hendrickx, P.M.S., Gutmanas, A., Westbrook, J.D., Yang, H., Feng, Z., Baskaran, K., Berrisford, J.M., Hudson, B.P. *et al.* (2017) Validation of Structures in the Protein Data Bank. *Structure*, **25**, 1916-1927.

Figure legends

Figure 1. **Mechanisms of long dsRNA digestion by RNase III enzymes.** (**A**) Human Dicer recognizes the dsRNA termini with a 2-nucleotide 3′ overhang, cleaves both strands, and produces a duplex RNA of 22 nucleotides in each strand (The end-in mechanism). (**B**) An EcRNase III dimer recognizes the dsRNA termini, especially those featuring a 2-nucleotide 3′ overhang, cleaves both strands, and produces a short duplex RNA of 11 nucleotides in each strand (The end-in mechanism). Under special conditions, however, two EcRNase III dimers bind to and cleave dsRNA in a cooperative manner, which produces a duplex RNA of 22 nucleotides in each strand. (The inside-out mechanism). (**C**) Two EcEEQ (the E38A/E65A/Q165A triple mutant of EcRNase III) or EcE38A (the EcE38A single mutant of EcRNase III) dimers bind to and cleave dsRNA in a cooperative manner, which produces a duplex RNA of 22 nucleotides in each strand (The inside-out mechanism).

Figure 2. **Crystal Structure of the EcEEQ:RNA6 Complex**. (**A**) The two subunits of dimeric EcEEQ are shown in cyan and orange, respectively. RIIIDs are illustrated as molecular surfaces and dsRBDs as ribbon diagrams (helices as spirals, strands as arrows, and loops as tubes). RNA6 molecules are shown as cartoon models and Mg^{2+} ions as spheres. (**B**) The architecture of cleavage site is illustrated with nucleotide residues R-1, R 0 and R+1 (stick models, N in blue, C in green, O in red, and P in orange), 3 Mg^{2+} ions (spheres in black), and Mg^{2+}-coordinating water molecules (spheres in red) in one cleavage site assembly. The F_o - F_c omit map (contoured at 6.0 σ, in blue) is shown for the three Mg^{2+} ions. (**C**) Sequence alignment of EcRNase III (UniProtKB: P0A7Y0) and AaRNase III (UniProtKB: O67082) based on the crystal structures of EcEEQ:RNA6 (this work) and AaRNase III:RNA9 (PDB: 2NUG). Identical residues are

indicated with stars under the sequences. Helices are highlighted in green and strands in cyan. The three mutation sites are highlighted in red with residue numbers of EcRNase III shown above the sequences. Conserved residues are indicated with stars under the sequences and the four catalytic residues are highlighted in red.

Figure 3. **Structural Basis for the Function of EcEEQ, the Bacterial Dicer**. (**A**) Superposition of the EcEEQ:RNA6 (ribbon diagram in cyan and orange, this work) and AaRNase III:RNA9 (in gray, PDB: 2NUG) structures. Mg^{2+} ions in the EcEEQ:RNA6 structure are shown as black spheres to highlight the two RNA cleavage sites. Selected side chains are shown as stick models in atomic color scheme (N in blue, O in red, and C in cyan, orange, or gray). (**B**) Zoom-in view shows that the E38A and E65A mutations remove negative charges from the catalytic valley of EcRNase III, promoting the inside-out processing of long dsRNA. (**C**) Zoom-in view shows that the Q165 side chain forms three hydrogen bonds with the +3G nucleotide, two with the base and one with the 2′-OH group, and that the Q165A mutation abolishes the recognition of the +3G nucleotide by the enzyme. The length of the three hydrogen bonds ranges between 2.9 and 3.1 Å.

Figure 4. **Three Mg^{2+} Ions in the Postcleavage Complex of Bacterial RNase III**. (**A**) The catalytic site assembly of EcRNase III as observed in the EcEEQ:RNA6 structure (this work). Amino acid and nucleotide residues are illustrated as stick models, and Mg^{2+} ions and water oxygen atoms as spheres in atomic color scheme (C in cyan, N in blue, O in red, P in orange, and Mg in black). Solid lines indicate coordinate bonds. Dashed lines indicate hydrogen bonds. Double-arrowed lines indicate contact distances (CDs) between the 3′-oxygen of nucleotide R-1 and the phosphorus of nucleotide R 0 ($CD^{O3′,P}$) and between MgA and MgB ($CD^{MgA,MgB}$). (**B**)

The catalytic site assembly of AaRNase III as observed in the AaRNase III:RNA9 structure (PDB: 2NUG), exhibiting a minor conformation (25%, in atomic color scheme) and a major conformation (75%, in white). The MgC was not observed in the minor conformation most likely due to its low occupancy. For clarity, the four catalytic resides are not labeled in panel **B**.

Figure 5. **Stepwise Model for the Reaction Trajectory of Two-Mg^{2+}-Ion Catalysis by RNase III: Distinct Features between Prokaryotic and Eukaryotic Enzymes**. (**A-C**) Cleavage site architecture of bacterial RNase III at the postcleavage (structure), intermediate (model), and postcleavage (model) states are represented by the EcEEQ:RNA6 structure (this work) and models derived from the structure. The amino acid side chains and nucleotide residues are shown as stick models and Mg^{2+} ions and water oxygens as spheres (C in grey, N in blue, O in red, P in orange, and Mg in black). Metal co-ordination bonds are illustrated as solid lines and hydrogen bonds as dashed lines. The nucleotide residue in the middle is numbered 'R 0' and the rest are numbered according to the polarity of the RNA strand. (**D**) Structure-based sequence alignment of EcRNase III (this work), AaRNase III, (PDB: 2NUG), ScRnt1p (PDB: 4OOG), HsDrosha (PDB: 5B16), and HsDicer (PDB: 5ZAL). Conserved amino acid residues are highlighted in red. Residues E1, D2, D3, and E4 are conserved in all RNase IIIs, whereas N5 and K6 are unique for eukaryotic RNase IIIs. (**E-G**) Cleavage site architecture of yeast Rnt1p at the postcleavage (structure), intermediate (model), and postcleavage (model) states are represented by the structure of ScRnt1p postcleavage complex (PDB: 4OOG) and models derived from the structure, illustrated in the same manner as panels A-C except that the carbon atoms in residues N5 and K6 are highlighted in cyan.

Table 1. data collection and structure refinement statistics

**Data Collection**	
Space group	$P2_1$
Cell constants	
a, b, c (Å)	56.93, 65.75, 84.52
α, β, γ (°)	90.0, 102.09, 90.0
Resolution (Å)	30.00–1.80 (1.86–1.80)*
Completeness (%)	99.5 (98.4)
Total / Unique reflections	362165 / 55901
Redundancy	6.5 (6.1)
$I/\sigma(I)$	14.4 (3.3)
R_{merge}	0.121 (0.881)
R_{pim}	0.053 (0.504)
$CC_{1/2}$	80.6 (60.4)
**Refinement**	
Resolution (Å)	28.31–1.80 (1.90–1.80)
No. of reflections	55856 (7711)
R_{work} / R_{free}	0.171 / 0.202
No. of atoms / B-factors (Å^2)	
Protein	3617 / 26.4
RNA	1196 / 27.84
Water	418 / 32.6
Mg^{2+}, K^+, Cl^-	15 / 29.69
Ethylene glycol, Tris	72 / 40.50
R.m.s. deviations	
Bond lengths (Å)	0.008
Bond angles (°)	1.018
Ramachandran plot (%)	
Favored	98.88
Allowed	1.12
Outliers	0

*Values in parentheses are for the highest-resolution shell.

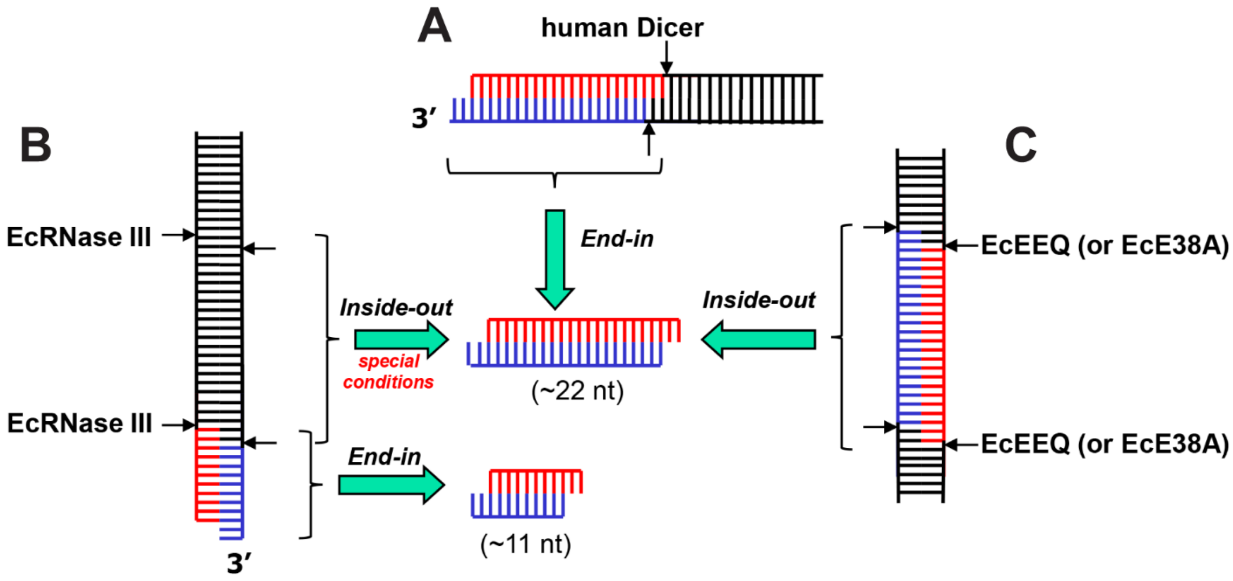

Figure 1. **Mechanisms of long dsRNA digestion by RNase III enzymes.** (**A**) Human Dicer recognizes the dsRNA termini with a 2-nucleotide 3' overhang, cleaves both strands, and produces a duplex RNA of 22 nucleotides in each strand (The end-in mechanism). (**B**) An EcRNase III dimer recognizes the dsRNA termini, especially those featuring a 2-nucleotide 3' overhang, cleaves both strands, and produces a short duplex RNA of 11 nucleotides in each strand (The end-in mechanism). Under special conditions, however, two EcRNase III dimers bind to and cleave dsRNA in a cooperative manner, which produces a duplex RNA of 22 nucleotides in each strand. (The inside-out mechanism). (**C**) Two EcEEQ (the E38A/E65A/Q165A triple mutant of EcRNase III) or EcE38A (the E38A single mutant of EcRNase III) dimers bind to and cleave dsRNA in a cooperative manner, which produces a duplex RNA of 22 nucleotides in each strand (The inside-out mechanism).

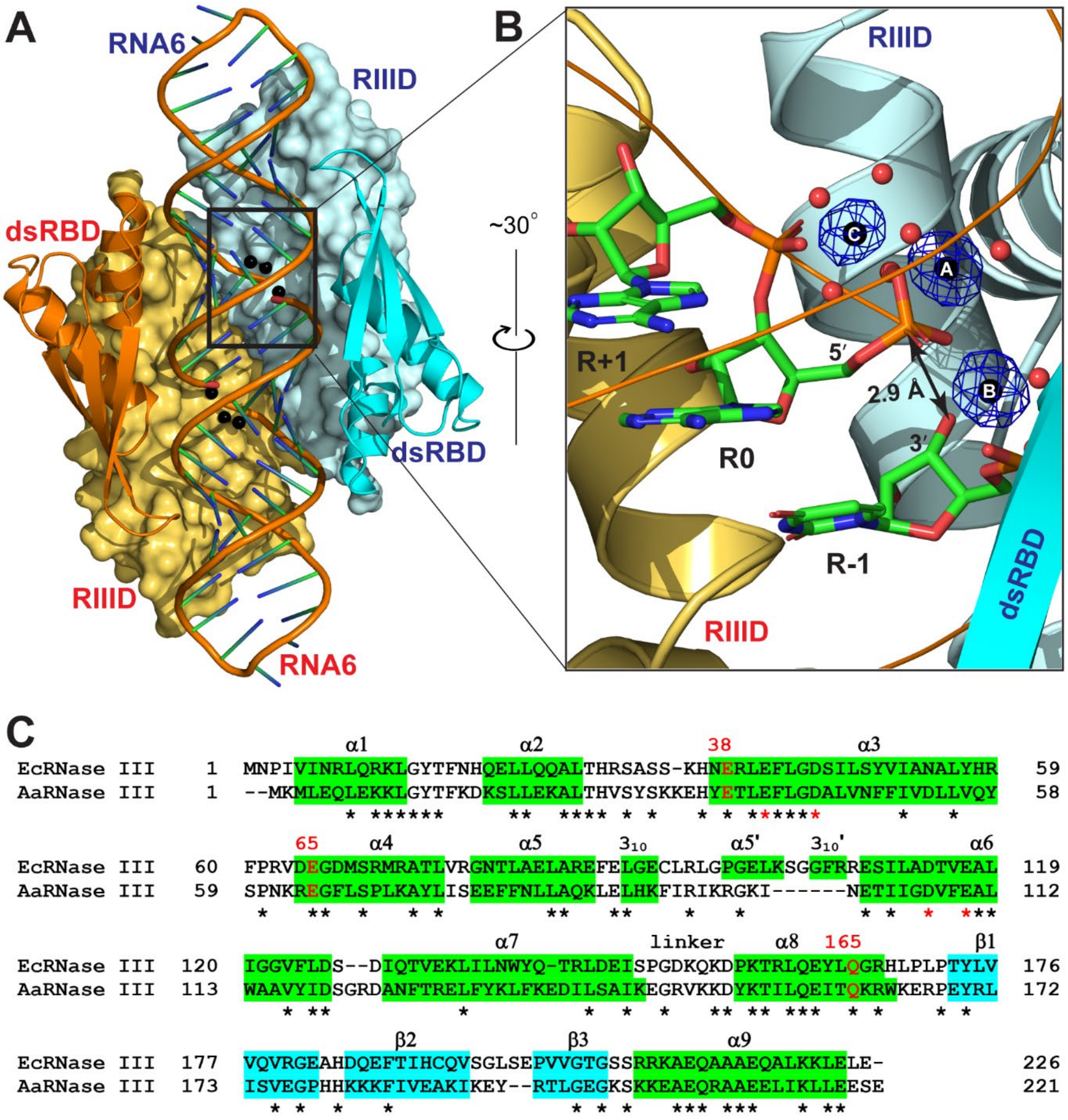

Figure 2. **Crystal Structure of the EcEEQ:RNA6 Complex.** (**A**) The two subunits of dimeric EcEEQ are shown in cyan and orange, respectively. RIIIDs are illustrated as molecular surfaces and dsRBDs as ribbon diagrams (helices as spirals, strands as arrows, and loops as tubes). RNA6 molecules are shown as cartoon models and Mg^{2+} ions as spheres. (**B**) The architecture of cleavage site is illustrated with nucleotide residues R-1, R 0 and R+1 (stick models, N in blue, C in green, O in red, and P in orange), 3 Mg^{2+} ions (spheres in black), and Mg^{2+}-coordinating water molecules (spheres in red) in one cleavage site assembly. The F_o - F_c omit map (contoured at 6.0 σ, in blue) is shown for the three Mg^{2+} ions. (**C**) Sequence alignment of EcRNase III (UniProtKB: P0A7Y0) and AaRNase III (UniProtKB: O67082) based on the crystal structures of EcEEQ:RNA6 (this work) and AaRNase III:RNA9 (PDB: 2NUG). Identical residues are indicated with stars under the sequences. Helices are highlighted in green and strands in cyan. The three mutation sites are highlighted in red with residue numbers of EcRNase III shown above the sequences. Conserved residues are indicated with stars under the sequences and the four catalytic residues are highlighted in red.

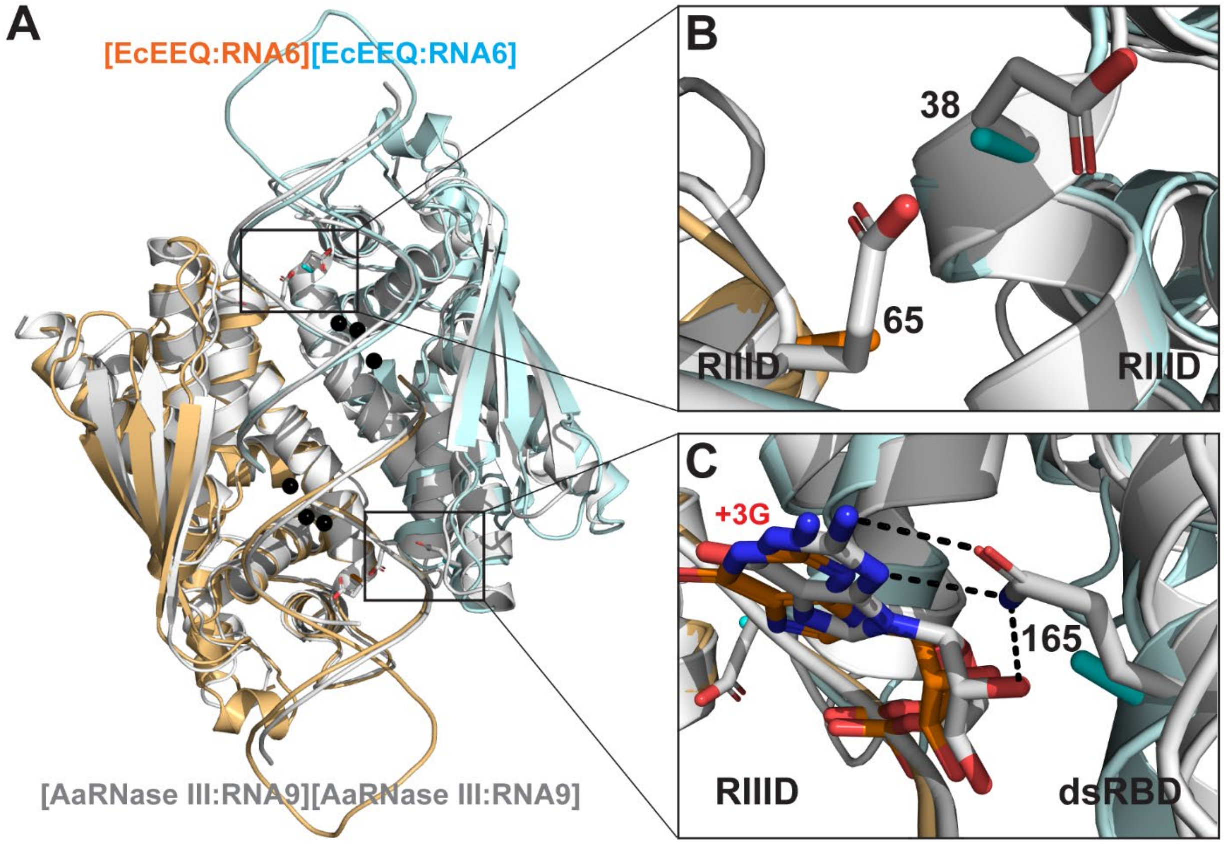

Figure 3. **Structural Basis for the Function of EcEEQ, the Bacterial Dicer**. (**A**) Superposition of the EcEEQ:RNA6 (ribbon diagram in cyan and orange, this work) and AaRNase III:RNA9 (in gray, PDB: 2NUG) structures. Mg^{2+} ions in the EcEEQ:RNA6 structure are shown as black spheres to highlight the two RNA cleavage sites. Selected side chains are shown as stick models in atomic color scheme (N in blue, O in red, and C in cyan, orange, or gray). (**B**) Zoom-in view shows that the E38A and E65A mutations remove negative charges from the catalytic valley of EcRNase III, promoting the inside-out processing of long dsRNA. (**C**) Zoom-in view shows that the Q165 side chain forms three hydrogen bonds with the +3G nucleotide, two with the base and one with the 2′-OH group, and that the Q165A mutation abolishes the recognition of the +3G nucleotide by the enzyme. The length of the three hydrogen bonds ranges between 2.9 and 3.1 Å.

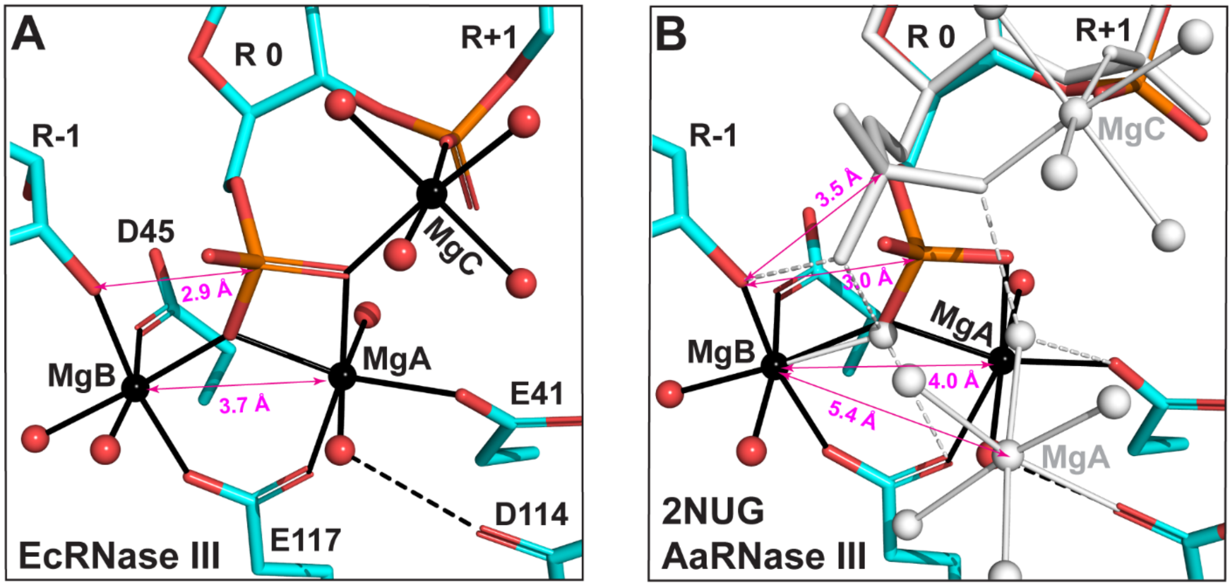

Figure 4. **Three Mg^{2+} Ions in the Postcleavage Complex of Bacterial RNase III.** (**A**) The catalytic site assembly of EcRNase III as observed in the EcEEQ:RNA6 structure (this work). Amino acid and nucleotide residues are illustrated as stick models, and Mg^{2+} ions and water oxygen atoms as spheres in atomic color scheme (C in cyan, N in blue, O in red, P in orange, and Mg in black). Solid lines indicate coordinate bonds. Dashed lines indicate hydrogen bonds. Double-arrowed lines indicate contact distances (CDs) between the 3'-oxygen of nucleotide R-1 and the phosphorus of nucleotide R 0 ($CD^{O3',P}$) and between MgA and MgB ($CD^{MgA,MgB}$). (**B**) The catalytic site assembly of AaRNase III as observed in the AaRNase III:RNA9 structure (PDB: 2NUG), exhibiting a minor conformation (25%, in atomic color scheme) and a major conformation (75%, in white). The MgC was not observed in the minor conformation most likely due to its low occupancy. For clarity, the four catalytic resides are not labeled in panel **B**.

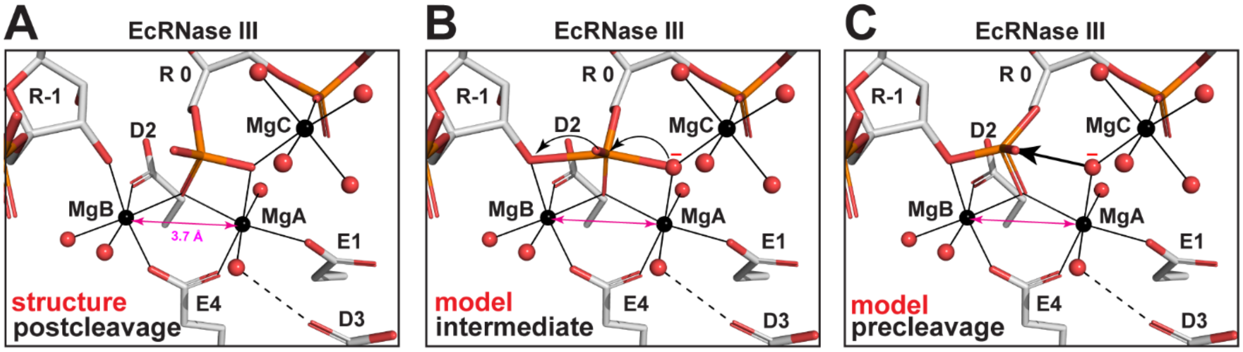

D

		E1 D2			N5			K6 D3 E4	
EcRNase III	38	ERLEFLGD	45	75	LVRGN	79	109	ESILADTVEA	118
AaRNase III	37	ETLEFLGD	44	74	LISEE	78	102	ETIIGDVFEA	111
ScRnt1p	238	ERLEFLGD	245	275	LVSNE	279	312	LKLYADVFEA	321
HsDrosha	1144	QRMEFLGD	1151	1181	LVNNR	1185	1214	TKTLADLLES	1223
HsDicer	1702	QRLEFLGD	1709	1739	LVNNT	1743	1805	PKAMGDIFES	1814

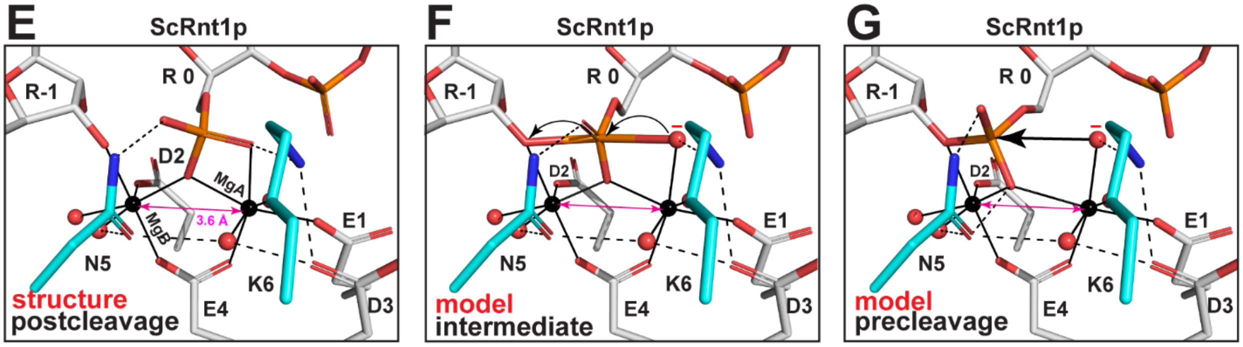

Figure 5. **Stepwise Model for the Reaction Trajectory of Two-Mg^{2+}-Ion Catalysis by RNase III: Distinct Features between Prokaryotic and Eukaryotic Enzymes**. (**A-C**) Cleavage site architecture of bacterial RNase III at the postcleavage, intermediate, and postcleavage states are represented by the EcEEQ:RNA6 structure (this work) and models derived from the structure. The amino acid side chains and nucleotide residues are shown as stick models and Mg^{2+} ions and water oxygens as spheres (C in grey, N in blue, O in red, P in orange, and Mg in black). Metal co-ordination bonds are illustrated as solid lines and hydrogen bonds as dashed lines. The nucleotide residue in the middle is numbered ‘R 0’ and the rest are numbered according to the polarity of the RNA strand. (**D**) Structure-based sequence alignment of EcRNase III (this work), AaRNase III, (PDB: 2NUG), ScRnt1p (PDB: 4OOG), HsDrosha (PDB: 5B16), and HsDicer (PDB: 5ZAL). Conserved amino acid residues are highlighted in red. Residues E1, D2, D3, and E4 are conserved in all RNase IIIs, whereas N5 and K6 are unique for eukaryotic RNase IIIs. (**E-G**) Cleavage site architecture of yeast Rnt1p at the postcleavage, intermediate, and postcleavage states are represented by the ScRnt1p:RNA structure (PDB: 4OOG) and models readily derived from the structure, illustrated in the same manner as panels A-C except that the carbon atoms in residues N5 and K6 are highlighted in cyan.
