# Supplementary material for: Structural basis for Dicer-like function of an engineered RNase III variant and insights into the reaction trajectory of two-Mg2+-ion catalysis
Source: RNA Biol. 2022 Jul 13;19(1):908–15. doi: 10.1080/15476286.2022.2099650 (PMC9291653; doi:10.1080/15476286.2022.2099650)
Supplement: Supplemental Material [file KRNB_A_2099650_SM4127.zip › 04_069_val_report_full_P1.pdf]

Preliminary Full wwPDB X-ray Structure Validation Report (i)

Mar 6, 2021 – 11:59 AM EST

This is a Preliminary Full wwPDB X-ray Structure Validation Report.

This report is produced by the standalone wwPDB validation server.
The structure in question has not been deposited to the wwPDB.
This report should not be submitted to journals.

We welcome your comments at *validation@mail.wwpdb.org*
A user guide is available at
https://www.wwpdb.org/validation/2017/XrayValidationReportHelp
with specific help available everywhere you see the (i) symbol.

PRELIMINARY VALIDATION REPORT

The following versions of software and data (see references (i)) were used in the production of this report:

MolProbity	:	4.02b-467
Mogul	:	1.8.5 (274361), CSD as541be (2020)
Xtriage (Phenix)	:	1.13
EDS	:	2.17.1
Percentile statistics	:	20191225.v01 (using entries in the PDB archive December 25th 2019)
Refmac	:	5.8.0158
CCP4	:	7.0.044 (Gargrove)
Ideal geometry (proteins)	:	Engh & Huber (2001)
Ideal geometry (DNA, RNA)	:	Parkinson et al. (1996)
Validation Pipeline (wwPDB-VP)	:	2.17.1

1 Overall quality at a glance (i)

The following experimental techniques were used to determine the structure:
X-RAY DIFFRACTION

The reported resolution of this entry is 1.80 Å.

Percentile scores (ranging between 0-100) for global validation metrics of the entry are shown in the following graphic. The table shows the number of entries on which the scores are based.

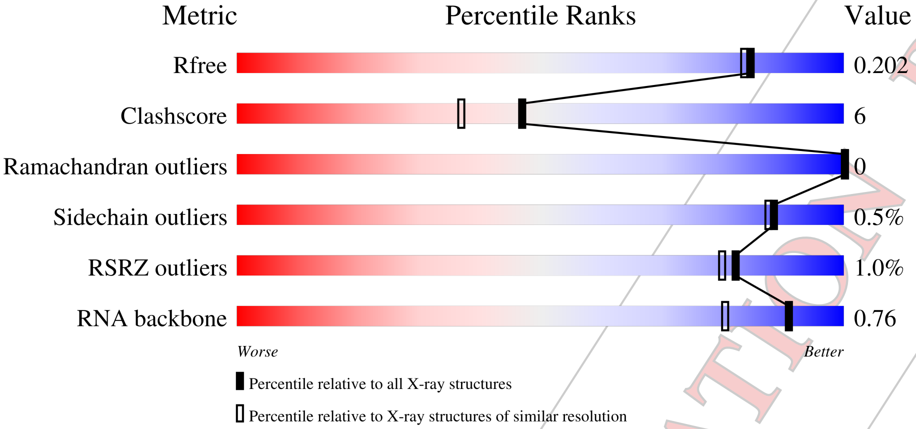

Metric	Whole archive (#Entries)	Similar resolution (#Entries, resolution range(Å))
R_{free}	130704	5950 (1.80-1.80)
Clashscore	141614	6793 (1.80-1.80)
Ramachandran outliers	138981	6697 (1.80-1.80)
Sidechain outliers	138945	6696 (1.80-1.80)
RSRZ outliers	127900	5850 (1.80-1.80)
RNA backbone	3102	1060 (2.40-1.20)

The table below summarises the geometric issues observed across the polymeric chains and their fit to the electron density. The red, orange, yellow and green segments of the lower bar indicate the fraction of residues that contain outliers for >=3, 2, 1 and 0 types of geometric quality criteria respectively. A grey segment represents the fraction of residues that are not modelled. The numeric value for each fraction is indicated below the corresponding segment, with a dot representing fractions <=5% The upper red bar (where present) indicates the fraction of residues that have poor fit to the electron density. The numeric value is given above the bar.

Mol	Chain	Length	Quality of chain
1	A	226	% / 90% 10%
1	B	226	92% 8%
2	C	28	4% / 75% 25%
2	D	28	4% / 71% 29%

PRELIMINARY VALIDATION REPORT

The following table lists non-polymeric compounds, carbohydrate monomers and non-standard residues in protein, DNA, RNA chains that are outliers for geometric or electron-density-fit criteria:

Mol	Type	Chain	Res	Chirality	Geometry	Clashes	Electron density
7	EDO	E	47	-	-	X	-

PRELIMINARY VALIDATION REPORT

2 Entry composition (i)

There are 8 unique types of molecules in this entry. The entry contains 5402 atoms, of which 84 are hydrogens and 0 are deuteriums.

In the tables below, the ZeroOcc column contains the number of atoms modelled with zero occupancy, the AltConf column contains the number of residues with at least one atom in alternate conformation and the Trace column contains the number of residues modelled with at most 2 atoms.

- Molecule 1 is a protein.

Mol	Chain	Residues	Atoms					ZeroOcc	AltConf	Trace
1	A	226	Total 1819	C 1137	N 333	O 344	S 5	0	5	0
1	B	226	Total 1798	C 1125	N 329	O 339	S 5	0	2	0

- Molecule 2 is a RNA chain called RNA (28-MER).

Mol	Chain	Residues	Atoms					ZeroOcc	AltConf	Trace
2	C	28	Total 598	C 267	N 106	O 197	P 28	0	0	0
2	D	28	Total 598	C 267	N 106	O 197	P 28	0	0	0

- Molecule 3 is MAGNESIUM ION (three-letter code: MG) (formula: Mg).

Mol	Chain	Residues	Atoms		ZeroOcc	AltConf
3	E	1	Total 1	Mg 1	0	0
3	E	1	Total 1	Mg 1	0	0
3	E	1	Total 1	Mg 1	0	0
3	E	1	Total 1	Mg 1	0	0
3	E	1	Total 1	Mg 1	0	0
3	E	1	Total 1	Mg 1	0	0

- Molecule 4 is POTASSIUM ION (three-letter code: K) (formula: K).

PRELIMINARY VALIDATION REPORT

Mol	Chain	Residues	Atoms	ZeroOcc	AltConf
4	E	1	Total K<br>1 1	0	0
4	E	1	Total K<br>1 1	0	0

- Molecule 5 is CHLORIDE ION (three-letter code: CL) (formula: Cl).

Mol	Chain	Residues	Atoms	ZeroOcc	AltConf
5	E	1	Total Cl<br>1 1	0	0
5	E	1	Total Cl<br>1 1	0	0
5	E	1	Total Cl<br>1 1	0	0
5	E	1	Total Cl<br>1 1	0	0
5	E	1	Total Cl<br>1 1	0	0
5	E	1	Total Cl<br>1 1	0	0
5	E	1	Total Cl<br>1 1	0	0

- Molecule 6 is 2-AMINO-2-HYDROXYMETHYL-PROPANE-1,3-DIOL (three-letter code: TRS) (formula: $C_4H_{12}NO_3$).

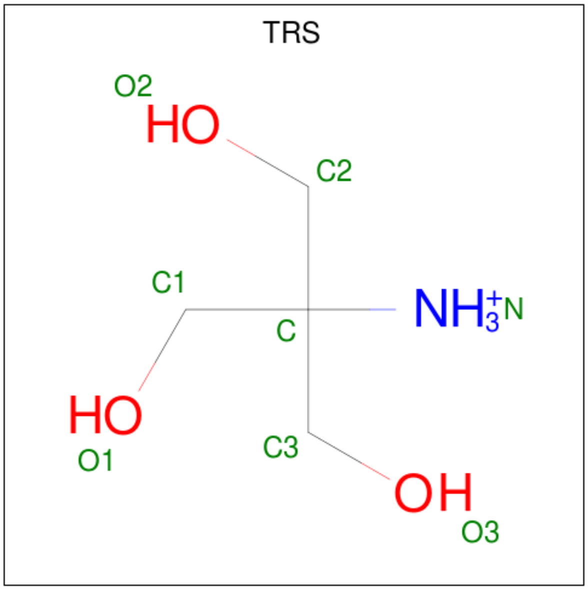

PRELIMINARY VALIDATION REPORT

Mol	Chain	Residues	Atoms	ZeroOcc	AltConf
6	E	1	Total C N O<br>8 4 1 3	0	0
6	E	1	Total C N O<br>8 4 1 3	0	0

- Molecule 7 is 1,2-ETHANEDIOL (three-letter code: EDO) (formula: $C_2H_6O_2$).

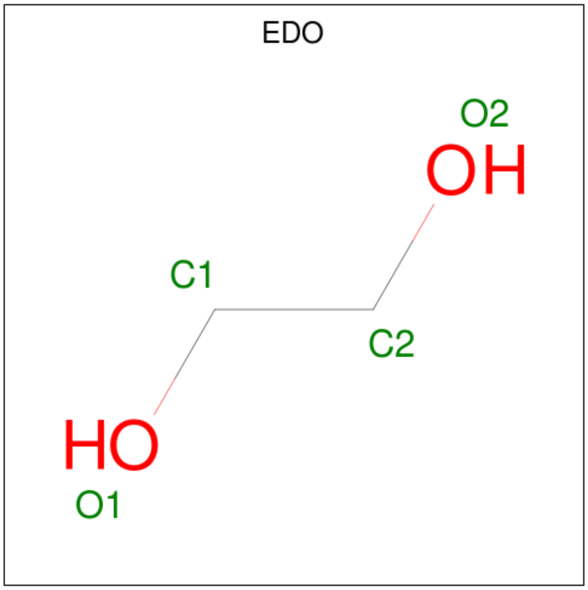

Mol	Chain	Residues	Atoms	ZeroOcc	AltConf
7	E	1	Total C H O<br>10 2 6 2	0	0
7	E	1	Total C H O<br>10 2 6 2	0	0
7	E	1	Total C H O<br>10 2 6 2	0	0
7	E	1	Total C H O<br>10 2 6 2	0	0
7	E	1	Total C H O<br>10 2 6 2	0	0
7	E	1	Total C H O<br>10 2 6 2	0	0
7	E	1	Total C H O<br>10 2 6 2	0	0
7	E	1	Total C H O<br>10 2 6 2	0	0
7	E	1	Total C H O<br>10 2 6 2	0	0
7	E	1	Total C H O<br>10 2 6 2	0	0

Continued on next page...

PRELIMINARY VALIDATION REPORT

Continued from previous page...

Mol	Chain	Residues	Atoms	ZeroOcc	AltConf
7	E	1	Total C H O<br>10 2 6 2	0	0
7	E	1	Total C H O<br>10 2 6 2	0	0
7	E	1	Total C H O<br>10 2 6 2	0	0
7	E	1	Total C H O<br>10 2 6 2	0	0

- Molecule 8 is water.

Mol	Chain	Residues	Atoms	ZeroOcc	AltConf
8	S	418	Total O<br>418 418	0	0

PRELIMINARY VALIDATION REPORT

3 Residue-property plots (i)

These plots are drawn for all protein, RNA, DNA and oligosaccharide chains in the entry. The first graphic for a chain summarises the proportions of the various outlier classes displayed in the second graphic. The second graphic shows the sequence view annotated by issues in geometry and electron density. Residues are color-coded according to the number of geometric quality criteria for which they contain at least one outlier: green = 0, yellow = 1, orange = 2 and red = 3 or more. A red dot above a residue indicates a poor fit to the electron density (RSRZ > 2). Stretches of 2 or more consecutive residues without any outlier are shown as a green connector. Residues present in the sample, but not in the model, are shown in grey.

- Molecule 1:

Chain A:

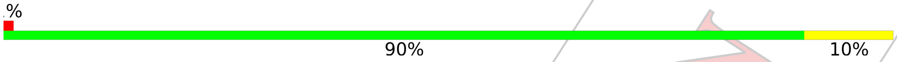

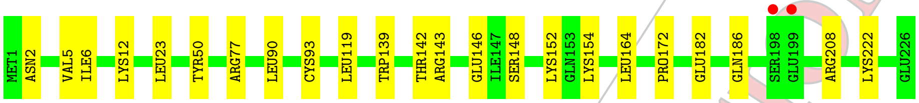

- Molecule 1:

Chain B:

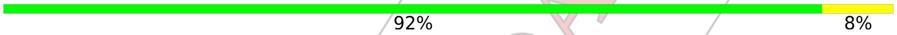

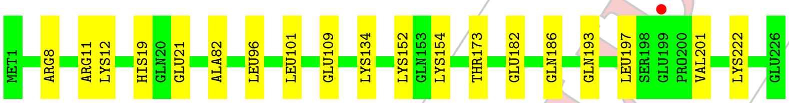

- Molecule 2: RNA (28-MER)

Chain C:

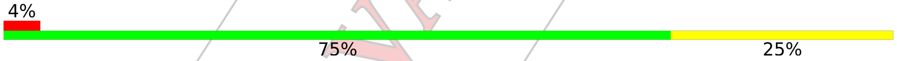

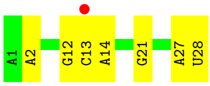

- Molecule 2: RNA (28-MER)

Chain D:

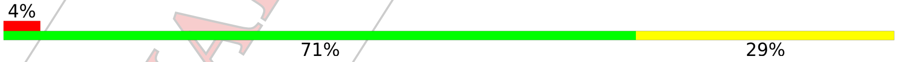

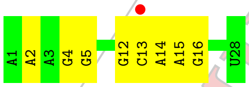

PRELIMINARY VALIDATION REPORT

4 Data and refinement statistics (i)

Property	Value	Source
Space group	P 1 21 1	Depositor
Cell constants<br>a, b, c, α, β, γ	56.93Å 65.75Å 84.52Å<br>90.00° 102.09° 90.00°	Depositor
Resolution (Å)	28.31 – 1.80<br>28.31 – 1.80	Depositor<br>EDS
% Data completeness<br>(in resolution range)	99.2 (28.31-1.80)<br>99.2 (28.31-1.80)	Depositor<br>EDS
R_{merge}	(Not available)	Depositor
R_{sym}	(Not available)	Depositor
$< I/\sigma(I) >$ [1]	3.85 (at 1.80Å)	Xtriage
Refinement program	phenix.refine 1.16_3549, PHENIX 1.16_3549	Depositor
R, R_{free}	0.171 , 0.202<br>0.171 , 0.202	Depositor<br>DCC
R_{free} test set	1000 reflections (1.79%)	wwPDB-VP
Wilson B-factor (Å^2)	23.0	Xtriage
Anisotropy	0.358	Xtriage
Bulk solvent k_{sol}(e/Å^3), B_{sol}(Å^2)	0.37 , 52.4	EDS
L-test for twinning[2]	$< \|L\| > = 0.49, < L^2 > = 0.32$	Xtriage
Estimated twinning fraction	No twinning to report.	Xtriage
F_o,F_c correlation	0.96	EDS
Total number of atoms	5402	wwPDB-VP
Average B, all atoms (Å^2)	27.0	wwPDB-VP

Xtriage's analysis on translational NCS is as follows: *The largest off-origin peak in the Patterson function is 7.43% of the height of the origin peak. No significant pseudotranslation is detected.*

[1] Intensities estimated from amplitudes.

[2] Theoretical values of $< |L| >$, $< L^2 >$ for acentric reflections are 0.5, 0.333 respectively for untwinned datasets, and 0.375, 0.2 for perfectly twinned datasets.

PRELIMINARY VALIDATION REPORT

5 Model quality (i)

5.1 Standard geometry (i)

Bond lengths and bond angles in the following residue types are not validated in this section: EDO, CL, K, TRS, MG

The Z score for a bond length (or angle) is the number of standard deviations the observed value is removed from the expected value. A bond length (or angle) with $|Z| > 5$ is considered an outlier worth inspection. RMSZ is the root-mean-square of all Z scores of the bond lengths (or angles).

Mol	Chain	Bond lengths		Bond angles	
		RMSZ	#$\|Z\|$ >5	RMSZ	#$\|Z\|$ >5
1	A	0.43	0/1851	0.56	0/2497
1	B	0.42	0/1830	0.56	0/2468
2	C	0.74	0/668	1.12	0/1037
2	D	0.76	0/668	1.09	0/1037
All	All	0.53	0/5017	0.76	0/7039

There are no bond length outliers.

There are no bond angle outliers.

There are no chirality outliers.

There are no planarity outliers.

5.2 Too-close contacts (i)

In the following table, the Non-H and H(model) columns list the number of non-hydrogen atoms and hydrogen atoms in the chain respectively. The H(added) column lists the number of hydrogen atoms added and optimized by MolProbity. The Clashes column lists the number of clashes within the asymmetric unit, whereas Symm-Clashes lists symmetry-related clashes.

Mol	Chain	Non-H	H(model)	H(added)	Clashes	Symm-Clashes
1	A	1819	0	1831	16	0
1	B	1798	0	1814	22	0
2	C	598	0	300	6	0
2	D	598	0	300	10	0
3	E	6	0	0	0	0
4	E	2	0	0	0	0
5	E	7	0	0	1	0
6	E	16	0	24	9	0
7	E	56	84	84	13	0

Continued on next page...

PRELIMINARY VALIDATION REPORT

Continued from previous page...

Mol	Chain	Non-H	H(model)	H(added)	Clashes	Symm-Clashes
8	S	418	0	0	6	0
All	All	5318	84	4353	57	0

The all-atom clashscore is defined as the number of clashes found per 1000 atoms (including hydrogen atoms). The all-atom clashscore for this structure is 6.

All (57) close contacts within the same asymmetric unit are listed below, sorted by their clash magnitude.

Atom-1	Atom-2	Interatomic distance (Å)	Clash overlap (Å)
1:B:96:LEU:H	7:E:47:EDO:H22	1.47	0.80
1:B:134:LYS:HE3	8:S:343:HOH:O	1.81	0.78
1:B:173:THR:OG1	1:B:193:GLN:HB3	1.84	0.76
1:A:154:LYS:HE2	8:S:278:HOH:O	1.87	0.73
1:B:154:LYS:HE2	8:S:274:HOH:O	1.88	0.73
6:E:31:TRS:H21	8:S:379:HOH:O	1.92	0.68
2:C:21:G:OP1	7:E:53:EDO:H11	1.96	0.66
1:B:96:LEU:N	7:E:47:EDO:H22	2.12	0.64
1:A:143:ARG:HG3	5:E:27:CL:CL	2.34	0.64
1:B:101:LEU:HD21	7:E:47:EDO:H11	1.80	0.64
1:B:201:VAL:HG22	1:B:222:LYS:NZ	2.13	0.63
1:B:8:ARG:NH1	1:B:11:ARG:HH21	1.97	0.62
1:B:19:HIS:HB3	1:B:21:GLU:OE2	2.00	0.61
1:A:139:TRP:HA	7:E:44:EDO:H12	1.85	0.58
2:D:12:G:N7	6:E:32:TRS:H22	2.19	0.57
1:B:82:ALA:CB	1:B:109[B]:GLU:HG3	2.34	0.57
2:C:12:G:N7	6:E:31:TRS:H12	2.18	0.57
1:A:2:ASN:O	1:A:5[A]:VAL:HG22	2.05	0.56
2:D:12:G:C8	6:E:32:TRS:H12	2.41	0.56
2:D:4:G:O6	7:E:54:EDO:H22	2.06	0.55
1:B:201:VAL:HG22	1:B:222:LYS:HZ2	1.71	0.55
2:D:12:G:N7	6:E:32:TRS:H12	2.23	0.54
2:D:12:G:N7	6:E:32:TRS:H31	2.23	0.53
7:E:52:EDO:H11	7:E:54:EDO:O2	2.08	0.53
6:E:32:TRS:H21	8:S:55:HOH:O	2.09	0.52
1:B:82:ALA:HB2	1:B:109[B]:GLU:HG3	1.92	0.52
1:B:96:LEU:H	7:E:47:EDO:C2	2.20	0.52
1:B:8:ARG:HD2	1:B:11:ARG:HH21	1.77	0.50
2:C:12:G:N7	6:E:31:TRS:H22	2.25	0.50
1:B:8:ARG:HD2	1:B:11:ARG:NH2	2.26	0.49
1:A:142:THR:O	1:A:146[B]:GLU:HG2	2.13	0.49
2:D:15:A:H2'	2:D:16:G:O4'	2.12	0.49

Continued on next page...

PRELIMINARY VALIDATION REPORT

Continued from previous page...

Atom-1	Atom-2	Interatomic distance (Å)	Clash overlap (Å)
2:D:5:G:N7	7:E:52:EDO:H12	2.27	0.49
1:B:8:ARG:HH11	1:B:11:ARG:HH21	1.59	0.49
1:B:21:GLU:H	1:B:21:GLU:CD	2.17	0.47
1:B:182:GLU:O	1:B:186:GLN:HG3	2.15	0.47
2:C:12:G:C8	6:E:31:TRS:H12	2.50	0.47
1:A:12:LYS:HE2	7:E:41:EDO:O2	2.15	0.46
1:A:164:LEU:HD12	1:A:172:PRO:HD3	1.97	0.46
1:A:77:ARG:HH21	7:E:51:EDO:H11	1.81	0.45
1:B:173:THR:HG1	1:B:193:GLN:HB3	1.81	0.44
2:D:12:G:N2	2:D:15:A:N7	2.50	0.44
1:A:93:CYS:SG	7:E:41:EDO:H22	2.58	0.43
1:B:12:LYS:HE3	1:B:12:LYS:HB3	1.68	0.43
2:D:5:G:N7	7:E:52:EDO:C1	2.82	0.43
1:A:148:SER:OG	1:A:152:LYS:HD2	2.19	0.42
1:A:182:GLU:O	1:A:186:GLN:HG3	2.20	0.42
1:B:197:LEU:HD23	1:B:197:LEU:HA	1.92	0.41
1:A:208:ARG:HH11	1:A:208:ARG:HD3	1.74	0.41
1:A:90:LEU:HD21	1:A:119:LEU:HD11	2.03	0.41
1:A:164:LEU:CD1	1:A:172:PRO:HD3	2.50	0.41
2:C:13:C:H2'	2:C:14:A:O4'	2.21	0.41
2:D:13:C:C5	2:D:14:A:C4	3.08	0.41
1:A:222:LYS:HA	1:A:222:LYS:HE2	2.02	0.41
1:A:6:ILE:HG23	1:A:23:LEU:HD21	2.03	0.41
1:B:193:GLN:NE2	8:S:310:HOH:O	2.54	0.41
2:C:27:A:O2'	2:C:28:U:H5'	2.22	0.40

There are no symmetry-related clashes.

5.3 Torsion angles (i)

5.3.1 Protein backbone (i)

In the following table, the Percentiles column shows the percent Ramachandran outliers of the chain as a percentile score with respect to all X-ray entries followed by that with respect to entries of similar resolution.

The Analysed column shows the number of residues for which the backbone conformation was analysed, and the total number of residues.

Mol	Chain	Analysed	Favoured	Allowed	Outliers	Percentiles	
1	A	229/226 (101%)	227 (99%)	2 (1%)	0	100	100

Continued on next page...

PRELIMINARY VALIDATION REPORT

Continued from previous page...

Mol	Chain	Analysed	Favoured	Allowed	Outliers	Percentiles	
1	B	226/226 (100%)	223 (99%)	3 (1%)	0	100	100
All	All	455/452 (101%)	450 (99%)	5 (1%)	0	100	100

There are no Ramachandran outliers to report.

5.3.2 Protein sidechains ⓘ

In the following table, the Percentiles column shows the percent sidechain outliers of the chain as a percentile score with respect to all X-ray entries followed by that with respect to entries of similar resolution.

The Analysed column shows the number of residues for which the sidechain conformation was analysed, and the total number of residues.

Mol	Chain	Analysed	Rotameric	Outliers	Percentiles	
1	A	196/191 (103%)	195 (100%)	1 (0%)	88	87
1	B	193/191 (101%)	192 (100%)	1 (0%)	88	87
All	All	389/382 (102%)	387 (100%)	2 (0%)	88	87

All (2) residues with a non-rotameric sidechain are listed below:

Mol	Chain	Res	Type
1	A	50	TYR
1	B	152	LYS

Sometimes sidechains can be flipped to improve hydrogen bonding and reduce clashes. All (1) such sidechains are listed below:

Mol	Chain	Res	Type
1	A	178	GLN

5.3.3 RNA ⓘ

Mol	Chain	Analysed	Backbone Outliers	Pucker Outliers
2	C	27/28 (96%)	1 (3%)	0
2	D	27/28 (96%)	1 (3%)	0
All	All	54/56 (96%)	2 (3%)	0

All (2) RNA backbone outliers are listed below:

PRELIMINARY VALIDATION REPORT

Mol	Chain	Res	Type
2	C	2	A
2	D	2	A

There are no RNA pucker outliers to report.

5.4 Non-standard residues in protein, DNA, RNA chains (i)

There are no non-standard protein/DNA/RNA residues in this entry.

5.5 Carbohydrates (i)

There are no monosaccharides in this entry.

5.6 Ligand geometry (i)

Of 31 ligands modelled in this entry, 15 are monoatomic - leaving 16 for Mogul analysis.

In the following table, the Counts columns list the number of bonds (or angles) for which Mogul statistics could be retrieved, the number of bonds (or angles) that are observed in the model and the number of bonds (or angles) that are defined in the Chemical Component Dictionary. The Link column lists molecule types, if any, to which the group is linked. The Z score for a bond length (or angle) is the number of standard deviations the observed value is removed from the expected value. A bond length (or angle) with $|Z| > 2$ is considered an outlier worth inspection. RMSZ is the root-mean-square of all Z scores of the bond lengths (or angles).

Mol	Type	Chain	Res	Link	Bond lengths			Bond angles		
					Counts	RMSZ	$\#\|Z\| > 2$	Counts	RMSZ	$\#\|Z\| > 2$
7	EDO	E	41	-	3,3,3	0.53	0	2,2,2	0.15	0
6	TRS	E	32	-	7,7,7	0.36	0	9,9,9	0.61	0
7	EDO	E	51	-	3,3,3	0.48	0	2,2,2	0.30	0
7	EDO	E	47	-	3,3,3	0.52	0	2,2,2	0.23	0
7	EDO	E	49	-	3,3,3	0.48	0	2,2,2	0.25	0
7	EDO	E	42	-	3,3,3	0.51	0	2,2,2	0.26	0
7	EDO	E	48	-	3,3,3	0.48	0	2,2,2	0.42	0
7	EDO	E	50	-	3,3,3	0.46	0	2,2,2	0.22	0
7	EDO	E	43	-	3,3,3	0.46	0	2,2,2	0.27	0
7	EDO	E	45	-	3,3,3	0.51	0	2,2,2	0.22	0
7	EDO	E	54	4	3,3,3	0.54	0	2,2,2	0.16	0
6	TRS	E	31	-	7,7,7	0.38	0	9,9,9	0.49	0
7	EDO	E	44	-	3,3,3	0.41	0	2,2,2	0.55	0
7	EDO	E	53	-	3,3,3	0.47	0	2,2,2	0.25	0
7	EDO	E	46	-	3,3,3	0.55	0	2,2,2	0.05	0
7	EDO	E	52	-	3,3,3	0.47	0	2,2,2	0.10	0

PRELIMINARY VALIDATION REPORT

In the following table, the Chirals column lists the number of chiral outliers, the number of chiral centers analysed, the number of these observed in the model and the number defined in the Chemical Component Dictionary. Similar counts are reported in the Torsion and Rings columns. '-' means no outliers of that kind were identified.

Mol	Type	Chain	Res	Link	Chirals	Torsions	Rings
7	EDO	E	41	-	-	0/1/1/1	-
6	TRS	E	32	-	-	3/9/9/9	-
7	EDO	E	51	-	-	1/1/1/1	-
7	EDO	E	47	-	-	1/1/1/1	-
7	EDO	E	49	-	-	1/1/1/1	-
7	EDO	E	42	-	-	0/1/1/1	-
7	EDO	E	48	-	-	0/1/1/1	-
7	EDO	E	50	-	-	0/1/1/1	-
7	EDO	E	43	-	-	1/1/1/1	-
7	EDO	E	45	-	-	1/1/1/1	-
7	EDO	E	54	4	-	1/1/1/1	-
6	TRS	E	31	-	-	2/9/9/9	-
7	EDO	E	44	-	-	0/1/1/1	-
7	EDO	E	53	-	-	0/1/1/1	-
7	EDO	E	46	-	-	0/1/1/1	-
7	EDO	E	52	-	-	1/1/1/1	-

There are no bond length outliers.

There are no bond angle outliers.

There are no chirality outliers.

All (12) torsion outliers are listed below:

Mol	Chain	Res	Type	Atoms
6	E	31	TRS	N-C-C3-O3
7	E	52	EDO	O1-C1-C2-O2
6	E	31	TRS	C1-C-C3-O3
7	E	51	EDO	O1-C1-C2-O2
6	E	32	TRS	N-C-C2-O2
7	E	45	EDO	O1-C1-C2-O2
7	E	47	EDO	O1-C1-C2-O2
7	E	43	EDO	O1-C1-C2-O2
7	E	54	EDO	O1-C1-C2-O2
6	E	32	TRS	C1-C-C2-O2
6	E	32	TRS	C3-C-C2-O2
7	E	49	EDO	O1-C1-C2-O2

There are no ring outliers.

PRELIMINARY VALIDATION REPORT

9 monomers are involved in 22 short contacts:

Mol	Chain	Res	Type	Clashes	Symm-Clashes
7	E	41	EDO	2	0
6	E	32	TRS	5	0
7	E	51	EDO	1	0
7	E	47	EDO	4	0
7	E	54	EDO	2	0
6	E	31	TRS	4	0
7	E	44	EDO	1	0
7	E	53	EDO	1	0
7	E	52	EDO	3	0

5.7 Other polymers (i)

There are no such residues in this entry.

5.8 Polymer linkage issues (i)

There are no chain breaks in this entry.

PRELIMINARY VALIDATION REPORT

6 Fit of model and data (i)

6.1 Protein, DNA and RNA chains (i)

In the following table, the column labelled '#RSRZ> 2' contains the number (and percentage) of RSRZ outliers, followed by percent RSRZ outliers for the chain as percentile scores relative to all X-ray entries and entries of similar resolution. The OWAB column contains the minimum, median, 95th percentile and maximum values of the occupancy-weighted average B-factor per residue. The column labelled 'Q< 0.9' lists the number of (and percentage) of residues with an average occupancy less than 0.9.

Mol	Chain	Analysed	<RSRZ>	#RSRZ>2			OWAB(Å^2)	Q<0.9
1	A	226/226 (100%)	-0.31	2 (0%)	84	82	13, 23, 40, 51	0
1	B	226/226 (100%)	-0.17	1 (0%)	92	90	14, 26, 43, 59	0
2	C	28/28 (100%)	-0.48	1 (3%)	42	37	19, 23, 55, 67	0
2	D	28/28 (100%)	-0.45	1 (3%)	42	37	19, 21, 58, 70	0
All	All	508/508 (100%)	-0.26	5 (0%)	82	80	13, 24, 43, 70	0

All (5) RSRZ outliers are listed below:

Mol	Chain	Res	Type	RSRZ
2	D	13	C	3.4
2	C	13	C	3.1
1	A	199	GLU	2.7
1	A	198[A]	SER	2.2
1	B	199	GLU	2.0

6.2 Non-standard residues in protein, DNA, RNA chains (i)

There are no non-standard protein/DNA/RNA residues in this entry.

6.3 Carbohydrates (i)

There are no monosaccharides in this entry.

6.4 Ligands (i)

In the following table, the Atoms column lists the number of modelled atoms in the group and the number defined in the chemical component dictionary. The B-factors column lists the minimum,

PRELIMINARY VALIDATION REPORT

median, 95th percentile and maximum values of B factors of atoms in the group. The column labelled 'Q< 0.9' lists the number of atoms with occupancy less than 0.9.

Mol	Type	Chain	Res	Atoms	RSCC	RSR	B-factors(Å^2)	Q<0.9
6	TRS	E	31	8/?	0.53	0.33	45,52,54,66	0
6	TRS	E	32	8/?	0.69	0.25	42,45,55,57	0
7	EDO	E	54	4/?	0.80	0.17	27,36,60,60	0
7	EDO	E	49	4/?	0.84	0.19	30,39,49,52	0
7	EDO	E	53	4/?	0.85	0.21	34,43,51,61	0
7	EDO	E	44	4/?	0.87	0.14	33,41,50,58	0
7	EDO	E	52	4/?	0.87	0.17	25,33,44,53	0
7	EDO	E	41	4/?	0.89	0.13	41,54,60,67	0
7	EDO	E	45	4/?	0.90	0.11	36,43,44,48	0
7	EDO	E	43	4/?	0.90	0.18	38,50,63,65	0
7	EDO	E	51	4/?	0.90	0.11	29,48,51,57	0
7	EDO	E	47	4/?	0.91	0.12	31,38,60,63	0
3	MG	E	6	1/?	0.91	0.09	29,29,29,29	0
7	EDO	E	50	4/?	0.92	0.22	30,36,43,43	0
7	EDO	E	46	4/?	0.93	0.18	29,35,44,44	0
5	CL	E	23	1/?	0.93	0.11	55,55,55,55	0
3	MG	E	4	1/?	0.94	0.07	22,22,22,22	0
7	EDO	E	42	4/?	0.96	0.10	27,33,47,47	0
3	MG	E	3	1/?	0.96	0.04	28,28,28,28	0
7	EDO	E	48	4/?	0.96	0.16	22,27,31,35	0
3	MG	E	1	1/?	0.97	0.09	20,20,20,20	0
5	CL	E	24	1/?	0.97	0.04	35,35,35,35	0
3	MG	E	5	1/?	0.98	0.04	20,20,20,20	0
5	CL	E	25	1/?	0.98	0.05	32,32,32,32	0
5	CL	E	26	1/?	0.99	0.04	31,31,31,31	0
5	CL	E	27	1/?	0.99	0.09	29,29,29,29	1
5	CL	E	22	1/?	0.99	0.13	39,39,39,39	0
3	MG	E	2	1/?	0.99	0.03	18,18,18,18	0
4	K	E	12	1/?	0.99	0.05	24,24,24,24	0
5	CL	E	21	1/?	0.99	0.04	28,28,28,28	0
4	K	E	11	1/?	1.00	0.03	26,26,26,26	0

6.5 Other polymers (i)

There are no such residues in this entry.

PRELIMINARY VALIDATION REPORT
